# Association Between the Vascular Ageing of the Carotid Arteries and Different Dialysis Modalities

**DOI:** 10.7759/cureus.89519

**Published:** 2025-08-06

**Authors:** Boban Labovic, Violeta Rabrenovic, Milica Petrovic, Dejan Pilcevic, Toplica Lepic, Nemanja Rancic

**Affiliations:** 1 Clinic of Neurology, Military Medical Academy, Belgrade, SRB; 2 Clinic of Nephrology, Military Medical Academy, Belgrade, SRB; 3 Center for Clinical Pharmacology, Medical Faculty of Military Medical Academy, Belgrade, SRB; 4 Institute of Radiology, Medical Faculty of Military Medical Academy, Belgrade, SRB

**Keywords:** carotid atherosclerotic disease, color doppler ultrasound, hemodialysis access, peritoneal dialysis (pd), vascular age

## Abstract

Background

Chronic kidney disease is a major global health problem, significantly increasing the risk of cardiovascular complications compared to the general population. This study aimed to evaluate vascular aging in patients with end-stage renal disease treated with hemodialysis or peritoneal dialysis by analyzing clinical and laboratory parameters and carotid intima-media thickness (CIMT), with the goal of identifying predictors of carotid disease and comparing the prevalence between groups.

Methods

We conducted a retrospective cohort study that included 59 subjects with end-stage renal disease who were treated with renal replacement therapy: peritoneal dialysis (29 subjects) and hemodialysis (30 subjects).

Results

When observing these parameters collectively, it was noted that at least one of the parameters (intima-media thickness greater than 0.7 mm or stenosis of the carotid arteries) was statistically more frequently present in the group of patients treated with the peritoneal dialysis method compared to those treated with the hemodialysis method (72.4% vs. 46.7%; p=0.044). The correlation analysis showed that pathological findings on the carotid blood vessels statistically significantly correlated with assignment to the group treated with peritoneal dialysis (r=-0.262, p=0.045). According to the results of multivariate logistic regression analysis, the independent factor associated with carotid disease belonged to the peritoneal dialysis group (odds ratio=3.24; 95% confidence interval=1.00-13.47). Subjects treated with peritoneal dialysis had a 3.244 times greater chance of developing carotid disease.

Conclusion

Monitoring clinical and laboratory parameters and changes in carotid blood vessels is important in patients with chronic kidney disease to prevent carotid atherosclerotic disease and other cardiovascular diseases. In our study, patients undergoing peritoneal dialysis had a 3.244 times higher likelihood of developing carotid disease. A careful selection of dialysis modality may influence the slowing of carotid atherosclerotic disease progression.

## Introduction

Atherosclerosis, a process of thickening and damage to the blood vessel wall, is the most common asymptomatic complication already present in the early stages of chronic kidney disease (CKD). CKD is present in around 10% of the world’s population [1].

Atherosclerosis is a significant predictor of serious cardiovascular diseases (CVDs) (cerebral and myocardial ischemia, heart valve disease, hypertensive heart disease, arrhythmias, and many others), especially in patients with end-stage renal failure [2]. The risk for CVD is 10-fold higher in patients with CKD compared to the general population [3]. CVDs are considered to be responsible for almost 50% of mortality in dialysis patients [4]. Atherosclerosis includes subclinical lesions manifested as increased wall thickness (intima and media) of the carotid artery and more advanced lesions in the form of plaque and stenosis [5]. The carotid intima-media thickness (CIMT) is the first sign of atherosclerotic vascular disease, which is followed by the deposition of plaques and the development of lumen stenosis. It gives a picture of the changes caused by the action of numerous risk factors on the arterial walls [6]. The correlation between CIMT and many cardiovascular risk factors is significant, especially in patients with chronic renal failure, and its early detection enables a timely therapeutic approach and the possibility of slowing the progression of kidney disease [7]. Determination of CIMT in patients with end-stage renal failure who were treated with renal function replacement (hemodialysis [HD] and peritoneal dialysis [PD]) enables the evaluation of the clinical pattern and prevalence of CVD in this patient group. In some studies, it was shown that the frequency of atherosclerotic plaques was statistically, significantly higher in patients treated with HD; in others, it was higher in patients treated with PD [8,9], while some studies do not observe statistically significant differences and an independent correlation [10].

This study aimed to evaluate vascular aging in patients with end-stage renal disease treated with HD or PD by analyzing clinical and laboratory parameters and CIMT, with the goal of identifying predictors of carotid disease and comparing the prevalence between groups.

## Materials and methods

A retrospective cohort study included 59 subjects, over 18 years old, with an average age of 53.71 ± 13.42 years, both sexes, with end-stage renal disease, who were treated with renal replacement therapy (PD and HD). The first group (29 subjects) consisted of patients treated with PD (continuous ambulatory peritoneal dialysis modality), and the second group (30 subjects) consisted of patients treated with HD (three times a week for 240 minutes).

The inclusion criteria in this study were as follows: patients with end-stage renal disease on regular HD and PD, who gave written consent for participating in the study; end-stage renal disease was defined as chronic advanced kidney disease in which the glomerular filtration rate was less than 15 mL/min/1.73m^2^; age above 18 years; at least six months' duration of dialysis; the absence of cardiovascular events three months before the study; and the absence of acute infection signs.

The following clinical data were monitored and compared in the subjects: body mass index (BMI), arterial blood pressure, and chosen laboratory parameters. Nonspecific inflammatory markers included C-reactive protein (CRP) (mg/L). Blood count parameters included erythrocytes (10^12^/L), hemoglobin (g/L), hematocrit (Hct) (L/L), leucocytes (10^9^/L), and thrombocytes (10^9^/L). Biochemical parameters included urea (mmol/L), creatinine (μmol/L), uric acid (μmol/L), glucose (mmol/L), total proteins (g/L), albumin (g/L), cholesterol (mmol/L), triglycerides (TG) (mmol/L), high-density lipoprotein (HDL) cholesterol (mmol/L), low-density lipoprotein (LDL) cholesterol (mmol/L), iron (μmol/L), calcium (mmol/L), and parathormone (PTH) (pmol/L). Transaminases were determined as well: alanine aminotransferase (ALT) (U/L), aspartate aminotransferase (AST) (U/L), alkaline phosphatase (ALP) (U/L), and lactate dehydrogenase (LDH) (U/L). The devices that were used to determine biochemical parameters were Auto-chemistry Analyzer DIRUI CS-300B (DIRUI Industrial Co., Ltd, Jilin, China) and Integrated chemistry system Dimension RXL MAX Siemens Healthineers (Siemens Healthcare GmbH, Erlangen, Germany). The devices for determining blood parameters were Hematology system ADVIA 120 Siemens (Siemens Healthineers, Forchheim, Germany) and Automatic hematology analyzer MACCURA F800 (Maccura Biotechnology Co., Ltd., Chengdu, China). UniCel DxI 800 Access Immunoassay System (Beckman Coulter, Inc., Brea, CA, USA) was used to determine PTH.

In all subjects, atherogenic index of plasma (AIP) was determined using the following formula: AIP = log TG/HDL. Patients were classified according to the AIP score: low risk (AIP < 0.11), intermediate risk (AIP 0.11-0.21), and high risk (AIP > 0.21).

Grayscale imaging or "B-mode" typically served as the initial step of examination where it evaluated the course and caliber of the vessel, including intimal-media thickness and plaque characteristics. Carotid arteries ware examined from the jugular notch to the angle of the mandible in transverse and longitudinal planes. Assessment included plaque echogenicity, surface characteristics, the presence of calcification, and stenosis size. At the jugular notch, the transducer was angled caudally, and, at the angle of the mandible, it was angled cephalically. Doppler examination was then performed. During the examination, the insonation angle was maintained at less than 60° to the axis of the carotid arteries. The sample volume was positioned at the center of the lumen and moved along the entire vessel for comprehensive evaluation. Spectral analysis included critical parameters, such as peak systolic and diastolic velocity, and pulsatility index. A color Doppler sonographic examination of the carotid blood vessels was performed on all patients in whom CIMT was measured. CIMT represents the distance between the most apparent echo obtained from the lumen-intima boundary line and the echo from the media-adventitia boundary line. The percentage of carotid lumen stenosis was also measured. CIMT assessment was conducted using ultrasound on a Toshiba Aplio 500 device with a PLT-704 SBT/7.5MHz B-mode probe (Toshiba, Minato, Japan). The criterion for CIMT was CIMT ≥ 0.7 mm, which presents a marker for atherosclerosis [11]. The carotid atherosclerotic disease was defined by the combined assessment of CIMT and the lumen of the carotid arteries stenosis, which indicated vascular aging. Carotid Doppler ultrasonography was performed by a single operator at the patients' bedside, and stenosis with CIMT was measured.

Carotid or vascular aging refers to the process of aging and the accumulation of plaque on the walls of the carotid arteries, which can lead to narrowing or occlusion of these blood vessels. The first sign of vascular aging is thickening of the intima-media complex. The second sign is the accumulation of plaque that leads to narrowing of the lumen of the arteries (stenosis). The third sign is micro-inflammation and oxidative stress that damage the walls of the blood vessels and accelerate the disease. All three mechanisms accelerate the development of CVD.

The Ethics Committee of the Military Medical Academy approved this study (No. 49/2024). The personal identifiers of the study participants were coded, and the medical data of the patients were anonymized and de-identified before analysis to preserve confidentiality. The study was conducted according to the principles of the Declaration of Helsinki.

Statistical analysis

A complete statistical analysis was conducted using the statistical program SPSS Version 26.0 (IBM Corp., Armonk, NY). The Kolmogorov-Smirnov test was used to check the normality of numerical data distribution. The significance of the difference in continuous variables was tested using the parametric Student’s t-test for independent samples if there was a normal distribution or using the Mann-Whitney test if the conditions for normal distribution were not met. Attributive data were presented in the form of absolute and relative frequencies, while the chi-square test was used to test the significance of differences between subgroups. Spearman’s correlation coefficient was used to determine correlations. Univariate logistic regression analysis (ULRA) was used to examine the association of demographic, clinical, laboratory, and ultrasound parameters with the dependent variable (carotid disease). All variables that were statistically significant in the univariate logistic regression model (significance level less than 0.05) were entered into the multivariate logistic regression analysis (MLRA) model with the aim of determining independent factors (predictors) associated with carotid disease.

## Results

In this study, a total of 59 dialysis patients were examined: 29 (49.2%) treated with PD and 30 (51.8%) treated with HD. Their demographic and clinical data are shown in Table 1.

**Table 1:** Demographic and clinical characteristics of the subjects (overall and in groups) Numerical data are presented as mean value ± standard deviation or as median value with interquartile range. Attributive data are shown as an absolute value (%). ^*^Independent samples test. ^#^Chi-square test. ^**^Mann-Whitney test. p<0.05 was considered statistically significant.

Characteristic	All subjects, n=59	Peritoneal dialysis, n=29	Hemodialysis, n=30	p-Value
Age (years)	53.71 ± 13.42	52.45 ± 13.76	54.93 ± 13.21	0.482^*^
Sex				
Male	25 (42.4)	13(44.8)	12(40.0)	0.911^#^
Female	34(57.6)	16(55.2)	18(60.0)	
Body mass index (kg/m^2^)	26.01 ± 4.07	26.35 ± 4.13	25.69 ± 4.06	0.538^*^
Systolic blood pressure (mmHg)	132.63 ± 19.93	134.66 ± 18.71	130.67 ± 21.18	0.447^*^
Diastolic blood pressure (mmHg)	76.90 ± 12.97	80.17 ± 11.76	73.73 ± 21.18	0.056^*^
Duration of dialysis (months)	36 (22-70)	38 (16.5-76)	36 (21-62.5)	0.952^**^

No statistically significant differences were observed in the demographic and clinical characteristics of patients treated with PD compared to those treated with HD. The average age of the subjects was 53.71 ± 13.42 years; in the group treated with PD, it was slightly lower (52.45 ± 13.76) compared to the group treated with HD (54.93 ± 13.21). Females dominated the overall distribution (57.6%), as well as in the groups: 55.2% in the PD group and 60% in the HD group. The median duration of dialysis in all subjects was 36 (22-70) months, with 38 (16.5-76) months in the PD group and 36 (21-62.5) months in the HD group.

Analysis of laboratory indicators between these two patient subgroups showed that patients treated with PD had significantly lower hemoglobin and hematocrit values compared to patients treated with HD (Table 2). Iron, calcium, and albumin levels were also significantly lower in patients treated with PD.

**Table 2:** Laboratory parameters of the subjects (overall and in groups) Numerical data are presented as mean value ± standard deviation or as median value with interquartile range. Attributive data are shown as an absolute value (%). *Independent samples test. #Mann-Whitney test. **Chi-square test. p<0.05 is considered statistically significant. PD, peritoneal dialysis; HD, hemodialysis; CRP, C-reactive protein; RBC, red blood cell (erythrocytes); Hb, hemoglobin; Hct, hematocrit; WBC, white blood cell (leukocytes); PLT, platelet (thrombocytes); Fe, iron; Glu, glucose; Ca, calcium; P, phosphorus; LDL, low-density lipoprotein cholesterol; HDL, high-density lipoprotein cholesterol; Trig, triglycerides; ALP, alkaline phosphatase; PTH, parathormone; ALT, alanine aminotransferase; AST, aspartate aminotransferase; LDH, lactate dehydrogenase; AIP, atherogenic index of plasma

Laboratory parameters	Reference range	All subjects	PD	HD	p-Value
CRP (mg/L)	0.00-4.00	3.82 (1.43-8.04)	4.50 (2.64-13.04)	1.72 (1.27-6.74)	0.102^#^
RBC (10^12^/L)	3.80-5.80	3.77±0.98	3.72± 1.29	3.82± 0.55	0.707^*^
Hb (g/L)	115-165	110.93±18.01	102.11± 15.70	119.47± 16.04	<0.001^*^
Hct (L/L)	0.37-0.47	0.34±0.05	0.32± 0.05	0.37± 0.05	<0.001^*^
WBC (10^9^/L)	4.00-11.00	7.05 ±2.62	7.66± 2.61	6.47± 2.54	0.079^*^
PLT (10^9^/L)	160-370	243.34± 76.36	243.45± 67.45	243.23± 85.27	0.991^*^
Fe (μmol/L)	9.-31	12.85± 5.78	11.14± 4.29	14.50± 6.59	0.025^*^
Glu (mmol/L)	4.1-5.9	5.40 (4.70-6.10)	5.40 (4.65-6.40)	5.65 (4.62-6.10)	0.994^#^
Urea (mmol/L)	2.5-7.5	22.52± 7.60	20.62± 8.75	24.36± 5.89	0.059^*^
Creatinine (μmol/L)	44-86	808 (690-1023)	884 (714-1029)	790 (667.25-996.25)	0.666^#^
Uric acid (μmol/L)	184-464	365.61± 71.16	348.86± 77.54	381.80± 61.41	0.075^*^
Albumin (g/L)	32-50	37.66± 3.57	36.41± 3.66	38.87± 3.09	0.007^*^
Total proteins (g/L)	60-83	63.08± 6.00	63.62± 6.14	62.57± 5.92	0.505^*^
Ca (mmol/L)	2.15-2.60	2.28± 0.20	2.22± 0.18	2.33± 0.20	0.049^*^
P (mmol/L)	0.78-1.65	1.75± 0.46	1.74± 0.46	1.77± 0.47	0.741^*^
Total cholesterol (mmol/L)	<5.2	5.04± 1.10	5.09± 1.34	4.99± 0.84	0.708^*^
LDL cholesterol (mmol/L)	<3.0	3.04± 0.84	3.16± 0.97	2.94± 0.70	0.324^*^
HDL cholesterol (mmol/L)	>1.5	1.38± 0.63	1.49± 0.76	1.28± 0.46	0.198^*^
Trig (mmol/L)	<1.7	2.12 (1.29-2.76)	2.11 (1.15-2.54)	2.37 (1.55-2.81)	0.249^#^
ALP (U/L)	70-290	170 (139-239)	185 (143-252.5)	158 (137.25-195.50)	0.126^#^
PTH (pmol/L)	1.30-9.30	41.20 (17.10-104.30)	50.00 (23.10-115.30)	31.65 (16.15-83.58)	0.143^#^
AST (U/L)	0-37	18.00 (13.00-24.00)	19.00 (12.00-24.50)	17.00 (14.75-22.00)	0.808^#^
ALT (U/L)	10-49	20.00 (16.00-26.00)	20.00 (14.50-25.50)	21.00 (16.00-28.25)	0.387^#^
LDH (U/L)	120-246	189 (170-232)	200 (171.5-271.5)	188 (164.75-218)	0.324^#^
AIP		0.25 (0.03-0.42)	0.17 (0.12-0.30)	0.29 (0.05-0.45)	0.063^#^
AIP groups					
Low risk	<0.11	21 (35.6)	12 (41.4)	9 (30.0)	0.157^**^
Intermediate risk	0.11-0.21	5 (8.5)	4 (13.8)	1 (3.3)	
High risk	>0.21	33 (55.9)	13 (44.8)	20 (66.7)	

Other laboratory parameters did not differ significantly between the subgroups. When AIP was observed, no statistically significant difference was found either, as the significance is borderline. However, it was evident that patients treated with HD had higher values, with their median being around 70% higher on average, which is clinically highly significant. When looking at the division into subgroups, we found a greater percentage of patients in the HD group at high risk compared to the PD group (66.7% vs. 44.8%).

A comparison of carotid blood vessel findings showed that the group of patients treated with HD exhibited more frequent intima-media thickness, while the group of patients treated with PD had a higher incidence of carotid artery stenosis (Table 3). When observing these parameters collectively, it was noted that at least one of the parameters (intima-media thickness greater than 0.7 mm or carotid artery stenosis) was statistically more prevalent in the group of patients treated with PD compared to the patients treated with HD (72.4% vs. 46.7%; p = 0.044).

**Table 3:** Carotid disease parameters: IMC thickness and carotid artery stenosis *Chi-square test. Attributive data is shown as an absolute value (%). p<0.05 is considered statistically significant. PD, peritoneal dialysis; HD, hemodialysis; IMC, intima-media complex

	All subjects	PD	HD	p-Value
IMC				
Normal findings	21 (35.6)	8 (27.6)	13 (43.3)	0.227^*^
Thickness < 0.7 mm	6 (10.2)	2 (6.9)	4 (13.3)	
Thickness ≥ 0.7 mm	32 (54.2)	19 (65.5)	13 (43.3)	
Stenosis				
No	44 (74.6)	20 (69.0)	24 (80.0)	0.500^*^
Yes	15 (25.4)	9 (31.0)	6 (20.0)	
Collective IMC ≥ 0.7 + stenosis				
Normal findings	24 (40.7)	8 (27.6)	16 (53.3)	0.044^*^
Pathological findings	35 (59.3)	21 (72.4)	14 (46.7)	

The correlation analysis showed that pathological findings in the carotid blood vessels statistically significantly correlated with belonging to the group of patients who underwent PD (r = -0.262, p = 0.045) (Table 4).

**Table 4:** Correlation between collective pathological findings in the carotid blood vessels and other significant variables Spearman’s correlation. p<0.05 is considered statistically significant. IMC, intima-media complex; AIP, atherogenic index of plasma; PD, peritoneal dialysis; HD, hemodialysis

		Collective IMC + stenosis
Dialysis duration	Correlation coefficient	0.192
	p-value	0.146
Age	Correlation coefficient	0.218
	p-value	0.097
AIP	Correlation coefficient	-0.011
	p-value	0.933
PD/HD	Correlation coefficient	-0.262
	p-value	0.045

According to the results of the ULRA, belonging to the PD-treated group was associated with a higher prevalence of carotid disease (odds ratio [OR] = 3.00; 95% confidence interval [CI] = 1.01-8.88).

When predicting the development of carotid disease in the form of stenosis or intima-media thickness, the results of the MLRA showed that an independent factor associated with carotid disease was belonging to the PD group (OR = 3.24; 95% CI = 1.00-13.47), meaning that patients undergoing PD had a 3.244 times higher likelihood of developing these findings in carotid arteries (Table 5).

**Table 5:** Factors associated with the occurrence of carotid artery disease among subjects (results of the ULRA and MLRA) p<0.05 is considered statistically significant. ULRA, univariate logistic regression analysis; MLRA, multivariate logistic regression analysis; OR, odds ratio; CI, confidence interval; PD, peritoneal dialysis; HD, hemodialysis; BMI, body mass index; CRP, C-reactive protein; Er, erythrocytes; Hb, hemoglobin; Hct, hematocrit; Le, leukocytes; Tr, thrombocytes; Fe, iron; Glu, glucose; Ca, calcium; P, phosphorus; LDL, low-density lipoprotein cholesterol; HDL, high-density lipoprotein cholesterol; Trig, triglycerides; AIP, atherogenic index of plasma; ALP, alkaline phosphatase; PTH, parathormone; AST, aspartate aminotransferase; ALT, alanine aminotransferase; LDH, lactate dehydrogenase; BP, blood pressure

	ULRA		MLRA	
	Crude OR (95% CI)	p-Value	Adjusted OR (95% CI)	p-Value
PD/HD	3.000 (1.014-8.880)	0.047	3.244 (1.004-13.468)	0.050
Sex	1.404 (0.486-4.054)	0.531	-	-
Age	1.032 (0.991-1.704)	0.130	-	-
Dialysis duration	1.016 (0.998-1.034)	0.079	-	-
BMI	0.984 (0.865-1.119)	0.805	-	-
CRP	0.982 (0.948-1.018)	0.330	-	-
Er	0.340 (0.124-0.932)	0.036	0.308 (0.024-4.025)	0.369
Hb	0.968 (0.938-0.999)	0.043	1.022 (0.931-1.123)	0.649
Hct	0.000 (0.000-0.357)	0.032	0.044 (0.000-0.001)	0.880
Le	0.914 (0.746-1.120)	0.384	-	-
Tr	0.997 (0.990-1.004)	0.398	-	-
Fe	1.035 (0.941-1.137)	0.479	-	-
Gluc	1.004 (0.876-1.149)	0.958	-	-
Urea	1.013 (0.944-1.086)	0.725	-	-
Creatinine	1.000 (0.998-1.003)	0.819	-	-
Uric acid	0.999 (0.992-1.007)	0.850	-	-
Albumin	0.856 (0.717-1.020)	0.082	-	-
Total proteins	0.971 (0.889-1.060)	0.507	-	-
Ca	0.428 (0.029-6.295)	0.536	-	-
P	2.197 (0.683-7.066)	0.187	-	-
Total cholesterol	0.871 (0.542-1.398)	0.567	-	-
LDL	1.107 (0.592-2.073)	0.750	-	-
HDL	0.585 (0.242-1.418)	0.235	-	-
Trig	1.066 (0.674-1.686)	0.784	-	-
AIP	1.090 (0.181-6.572)	0.925	-	-
AIP groups	0.982 (0.325-2.970)	0.640	-	-
ALP	1.001 (0.997-1.005)	0.684	-	-
PTH	1.004 (0.995-1.014)	0.334	-	-
AST	0.984 (0.951-1.019)	0.361	-	-
ALT	0.965 (0.926-1.006)	0.095	-	-
LDH	1.003 (0.994-1.011)	0.508	-	-
Systolic BP	1.009 (0.982-1.036)	0.521	-	-
Diastolic BP	1.038 (0.994-1.085)	0.095	-	-

## Discussion

Although it is known that patients with CKD are at an increased risk of developing CVD, numerous studies continue to investigate and classify various risk factors contributing to CVD in CKD patients. Identification and prevention of these factors can positively influence the slowing of carotid disease progression and the onset of other comorbidities. As arterial stiffness is a serious complication in end-stage renal disease, its measurement is crucial for disease prognosis and for planning interventions before irreversible vascular changes occur, especially in younger dialysis patients [7]. Additionally, the choice of treatment modality for end-stage renal disease, i.e., the type of dialysis used in the patient, is of great importance, given the conflicting results of previous studies regarding pro-atherosclerotic risk factors.

Our study was conducted on a homogeneous group of subjects treated with PD and HD, and the results did not show a statistically significant difference in demographic (age, sex) and clinical parameters (body mass index, systolic, diastolic blood pressure) or in the duration of the treatment procedures. This study design is similar to studies by other authors [11-15].

It is known that the increase in anemia is proportional to the decrease in kidney function [16,17]. One possible explanation for this can be the well-known phenomenon of eryptosis described in patients treated with PD, which implies the programmed death (apoptosis) of red blood cells [18]. Known triggers of eryptosis include an increased concentration of calcium (Ca) (2+) in the cytoplasm, oxidative stress, osmotic shock, exhaustion, and certain uremic toxins [18]. As a result of eryptosis, there is a reduced production of erythropoietin, a glycoprotein hormone that regulates the process of erythropoiesis and therefore prevents apoptosis. The relative lack of erythropoietin and the short life of erythrocytes are considered the most common causes of renal anemia [19-21].

In our study, a comparison of laboratory parameters between the two patient groups revealed a statistically significant relationship between treatment with PD and lower levels of erythrocytes, hemoglobin, and iron compared to patients undergoing HD.

Findings from other authors vary: some studies report no difference in the prevalence of anemia syndrome between these groups, while others indicate that PD patients predominantly experience iron deficiency [22,23].

Lower iron levels and a weaker response to erythropoiesis-stimulating agents (ESAs) in PD patients have also been reported in a Spanish study, which observed that HD patients receive intravenous iron supplementation more frequently and in higher doses compared to PD patients, who primarily receive oral iron. This explains the higher iron levels and better ESA response observed in HD patients compared to those undergoing PD in that study [24].

Some authors state the opposite: a less pronounced anemic syndrome in the group of patients treated with PD compared to those treated with HD, bearing in mind the specifics of the procedures themselves (increased risk of bleeding during HD as well as blood loss during the procedure itself). Thus, a group of Dutch authors states that in the group treated with HD, the risk of bleeding is 1.5 times higher compared to the risk in the group treated with PD (95% CI 1.0-2.2) [25].

In the meta-analysis by Wang et al., which included the results of 14 studies, no statistically significant difference was observed in the levels of hemoglobin and ferritin between these groups, but they indicated that the level of albumin was lower in the group treated with PD, similar to the results of our study [16]. Some studies describe the association of various laboratory parameters with cardiovascular comorbidity in dialysis patients: C-reactive protein and plasma fibrinogen [8,9], as well as the presence of metabolic syndrome [10].

Additionally, elevated levels of HDL and elevated atherogenic index of plasma were associated with increased thickness of the IMC and the presence of atherosclerotic plaques [11]. In our study, more frequent carotid artery stenosis was observed in patients treated with PD; additionally, it was identified that belonging to this dialysis group is an independent predictor of carotid disease, which aligns with findings from previous research [12-15]. Some studies describe opposite results, such as the study by Borras et al., which, in a study with 237 patients treated with PD and 451 treated with HD, found that CIMT was higher in the group treated with HD than in the group treated with PD. They suggest that PD is an independent risk factor associated with lower CIMT compared to HD [26].

It is known that glycoregulation disorder and dyslipidemia are predictors of atherosclerotic carotid disease [11,12]. Previous studies have shown that the frequency of metabolic syndrome is around 40% in HD patients and around 60% in PD patients and that the frequency of CVDs, including carotid disease, can be higher in patients with metabolic syndrome [10].

Glucose-based solutions that are used in PD significantly contribute to the development of obesity, metabolic syndrome, and inflammation in these patients compared to patients on HD [27]. The intraperitoneal use of glucose-based solutions in PD also contributes to increased oxidative stress and systemic effects of glucotoxicity, in addition to localized effects on the peritoneum and the development of peritoneal fibrosis. Prolonged use of these glucose-based solutions leads to functional and structural changes in the peritoneal membrane. Furthermore, the elevated concentration of glycation end-products in the skin of both diabetic and non-diabetic PD patients serves as evidence of systemic glucotoxicity, as described in previous studies [28].

Certain recent studies with longer monitoring of PD patients and monitoring of peritoneal glucose absorption and lipid status have shown no statistically significant effect on lipid profile (this has been attributed to the use of icodextrin and automated PD methods, as well as adjustments in peritoneal exchange prescriptions according to transport status) [29]. The results of the studies that included the issue of frequency and significance of cardiometabolic syndrome in patients treated with PD showed that dyslipidemia is manifested especially in the first year [23], which indicates the possible causes of the higher frequency of carotid atherosclerotic disease in this group of patients.

Many previous studies have described the association between greater intima-media thickness and cardiovascular risk factors, as well as the presence of CVD, including carotid disease [30,31]. It has also been shown that the intima-media thickness is an independent predictor of adverse cardiovascular events [31], which corresponds to the results of our study. The results of our study can contribute to the overall knowledge related to cardiovascular risks among end-stage renal disease patients on dialysis, as well as provide guidelines for future improvements in diagnostics and secondary prevention, bearing in mind that there are not many current studies dealing with this problem.

Nevertheless, this study has limitations related to the number of subjects and duration; however, we believe that it opens up interesting questions for further analyses that would clarify the results we obtained with the threshold values of significance. Another weakness of the study is its retrospective design. Therefore, further research with more subjects and longer monitoring in the prospective cohort study would be very useful to further investigate other factors significant for the development of carotid damage. Future studies should include and analyze confounders such as smoking and diabetes in order to better target patients and to obtain adequately stronger conclusions. Another limitation of this study is that confounders (e.g., diabetes, smoking) were not followed up.

## Conclusions

Monitoring laboratory and clinical parameters, as well as changes in blood vessels, is highly significant for patients with CKD in the prevention of carotid atherosclerotic disease and premature mortality. In our study, patients undergoing PD had a 3.244 times higher likelihood of developing carotid disease. A careful selection of dialysis modalities can slow the carotid atherosclerosis progression given that patients undergoing PD are at significantly higher risk of developing carotid disease compared to those on HD.
